# The effect of human wharton’s jelly-derived mesenchymal stem cells on MC4R, NPY, and LEPR gene expression levels in rats with streptozotocin-induced diabetes

**DOI:** 10.22038/IJBMS.2019.39582.9387

**Published:** 2020-02

**Authors:** Fatemeh Sabet Sarvestani, Mohammad Ali Zare, Forough Saki, Farhad Koohpeyma, Ismail H Al-Abdullah, Negar Azarpira

**Affiliations:** 1 Transplant Research Center, Shiraz University of Medical Sciences, Shiraz, Iran; 2 Endocrinology and Metabolism Research Center, Shiraz University of Medical Sciences, Shiraz, Iran; 3 Department of Translational Research and Cellular Therapeutics, Diabetes and Metabolism Research Institute, Beckman Research Institute of City of Hope, Duarte, USA

**Keywords:** Diabetes mellitus, Leptin, Melanocortin, Neuropeptide Y, Receptor, Stem cells, Wharton jelly

## Abstract

**Objective(s):**

Type 1 diabetes (T1D) is an autoimmune disease resulting from inflammatory destruction of islets β-cells. Nowadays, progress in cell therapy, especially mesenchymal stem cells (MSCs) proposes numerous potential remedies for T1D. We aimed to investigate the combination therapeutic effect of these cells with insulin and metformin on neuropeptide Y, melanocortin-4 receptor, and leptin receptor genes expression in TID.

**Materials and Methods:**

One hundreds male rats were randomly divided into seven groups: the control, diabetes, insulin (Ins.), insulin+metformin (Ins.Met.), Wharton’s Jelly-derived MSCs (WJ-MSCs), insulin+metformin+WJ-MSCs (Ins.Met.MSCs), and insulin+WJ-MSCs (Ins.MSCs). Treatment was performed from the first day after diagnosis as diabetes. Groups of the recipient WJ-MSCs were intraportally injected with 2× 10⁶ MSCs/kg at the 7th and 28th days of study. Fasting blood sugar was monitored and tissues and genes analysis were performed.

**Results:**

The blood glucose levels were slightly decreased in all treatment groups within 20^th^ and 45^th^ days compared to the diabetic group. The C-peptide level enhanced in these groups compared to the diabetic group, but this increment in Ins.MSCs group on the 45th days was higher than other groups. The expression level of melanocortin-4 receptor and leptin receptor genes meaningfully up-regulated in the treatment groups, while the expression of neuropeptide Y significantly down-regulated in the treatment group on both times of study.

**Conclusion:**

Our data exhibit that infusion of MSCs and its combination therapy with insulin might ameliorate diabetes signs by changing the amount of leptin and subsequent changes in the expression of neuropeptide Y and melanocortin-4 receptor.

## Introduction

Type 1 diabetes mellitus (T1DM) is an immune-mediated disorder that results from autoimmune destruction of pancreatic islet ß cells, which leads to little or no insulin production and uncontrolled high blood glucose levels. At the time of clinical diagnosis, 60-80% of pancreatic β-cells have been destroyed. Thus, regeneration of the endocrine pancreatic tissue is one of the strategies to treat T1DM (1).

Recently, stem cell therapy has achieved positive results in medicine related to the 3Rs (Replacement, Repair, and Regeneration). In fact, stem cell therapy has been applied successfully in diabetes, from preclinical to clinical studies (2-14). Mesenchymal stem cells (MSCs) are multipotent stromal cells with the capability of self-renewal and differentiation into a variety of cells (15). These cells have great potential, and it is feasible to isolate them; in addition, there is an abundant source, and ethical concerns are minimal. MSCs are isolated from various tissues, including adipose tissue, bone marrow, umbilical cord blood, umbilical cord, and dental pulp (16-18). When these cells are cultured *in vitro*, they appear as spindle-shaped cells. Furthermore, these cells express a specific marker profile; they are positive for CD29, CD51, CD73, CD90, and CD105 expression, while negative for hematopoietic markers such as CD31 and CD45 (19). Recently, MSCs have been considered as therapies for a wide range of diseases due to modulating immune responses and regenerative properties (20). Actually, human umbilical cord MSCs (hUC-MSCs) are adult stem cells, which are capable to differentiate into several cell phenotypes *in vitro* and *in vivo* (21). Interestingly, they do not express major histocompatibility complex (MHC) class II and only express MHC class I at low levels. Moreover, they do not express Fas ligand and co-stimulatory molecules such as B7 and CD40, thus they have been suggested to be hypoimmunogenic cells (22). Other properties include their ability to self-renew, to create colony forming units, and to differentiate into other cells such as bone, cartilage, and fat. The aforementioned characteristics are part of the criteria used to determine and identify MSCs (23). Moreover, MSCs can differentiate into myocytes, neuronal cells, and potentially, β-pancreatic islets cells. In some studies, it has been shown that MSCs have potential of differentiation into insulin-producing and islet-like cells (24-27). In addition to their differentiation potential, MSCs have been reported to regulate the immune response in many diseases (28-30). Maldonado *et al.* showed that human umbilical cord Wharton jelly cells (hUCWJCs) can migrate to damaged tissues and promote insulin secretion from non-pancreatic local cells (31). The differentiation potential and immunomodulatory feature of MSCs make these cells ideal candidates for the preservation of islet β cell function in patients with T1D (32).

Energy homeostasis is regulated by the interaction of nutrients and hormones with subpopulations of neurons located in several distinct areas of the brain. The hypothalamus, predominantly the arcuate nucleus (ARC), plays an important role in regulating food intake, energy expenditure and glucose metabolism. There are many factors to consider in the hypothalamic regulation of food intake, such as melanin-concentrating (MCH), Neuropeptide Y (NPY), agouti-related protein (AgRP), and proopiomelanocortin (POMC) (33).

**Figure 1:**
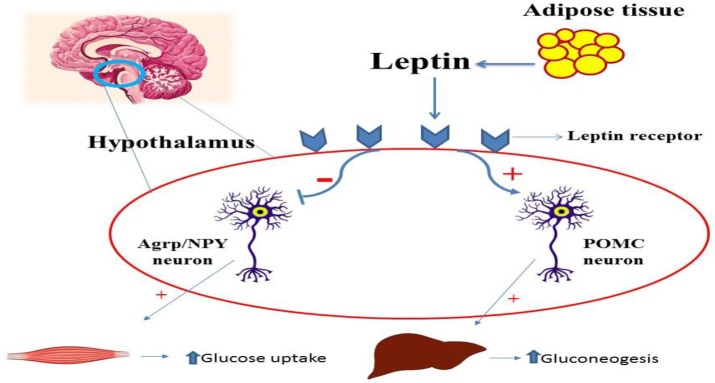
Leptin signaling pathways in CNS and peripheral tissues

Leptin (LEP) is a hormone, primarily derived from adipose tissue, with anti-diabetic features that regulates numerous physiological processes and behaviors, including appetite, body weight, neuroendocrine functions, and hypoglycemia. These actions are mediated through leptin receptors (LEPR) expressed by the nervous system (34). The POMC neurons in the ARC are activated by LEP. In fact, LEP is secreted by adipocytes, enters the CNS, and acts on its receptor expressed in key brain areas that regulate metabolism. This hormone inhibits NPY/AgRP neurons and stimulates POMC neurons in the ARC, responses to stimulation of glucose uptake in peripheral tissues and the suppression of glucose production from the liver. In addition, LEP induces gluconeogenesis via a melanocortin-dependent pathway (35-37) (Figure 1). Also, intracerebroventricular (ICV) infusing of LEP improved hyperglycemia, hyperglucagonemia, hyperketonemia, and polyuria caused by insulin deficiency in mice. Furthermore, ICV LEP delivery improves expression of the metabolically relevant hypothalamic neuropeptides POMC, NPY, and AgRP in T1D mice (38).

The aim of this study was to investigate the combination and segregation effects of WJ-MSCs, insulin, and metformin on blood glucose, C-peptide level, and expression of genes associated with metabolisms in T1D rat model. 

## Materials and Methods

***Animals, experimental groups, and diabetes induction***

One hundred adult male Sprague-Dawley rats (3-4 months old), weighing 200±20 g were used in the present randomized controlled experimental study. The rats were housed in the Center of Comparative and Experimental Medicine of Shiraz University of Medical Sciences, Shiraz, Iran, under controlled temperature (22 ˚C) and lighting (12 hr:12 hr light to dark ratio; light on at 7:30 AM) conditions. All experimental procedures on the rats were carried out between 7.00- 9.00 AM and based on the recommendations of the Animal Care Committee of the Shiraz University of Medical Sciences (SUMs) (Ethical code: IR.SUMS REC.1395.S673). In this experiment, a single dose (60 mg/kg) of Streptozotocin (STZ) (Sigma) was administered via the intraperitoneal (IP) cavity to develop T1D model (39, 40). Three weeks after STZ injection, fasting plasma glucose )FPG) of all rats were tested, and rat with blood glucose higher than 200 mg/dl was considered as diabetic. A few animals died during this period and were excluded from the study. Other animals were randomly divided into 7 groups: the control (n=10), diabetes (n=10), insulin (Ins.) (n=10), insulin+metformin (Ins.Met.) (n=10), MSC (n=10), Ins.Met.MSCs (n=10), and Ins.MSCs (n=10). Rats were treated everyday with insulin and metformin according to previously published protocols (41, 42) and received WJ-MSCs intraperitoneally twice with 2×10⁶ MSCs/kg at the first week and fourth weeks of study after diabetes confirmation according to the groups mentioned above (32). Daily fasting blood sugars were monitored in the treatment groups during the experiment. Finally, all rats were sacrificed to acquire blood and brain tissues for analysis on the 20^th^ and 45^th^ days of study.

***Isolation, Culture and Characterization of WJ-MSC***

Human umbilical cords (n=4) were collected from obstetric department affiliated to Shiraz University of Medical Sciences (SUMs), Shiraz, Iran. Informed consent was obtained from mothers, and the study was approved by our ethical committee of SUMS. Infectious pathology was excluded by the performance of human immunodeficiency virus (HIV), hepatitis C virus (HCV), and hepatitis B virus (HBV) tests. The tissues were stored in Hank’s balanced salt solution (HBSS; BiochromL201-10) containing antibiotics (penicillin 100 U/ml, streptomycin 100 μg/ml) on the ice. The isolation of MSCs was performed according to previously reported methods (43-45). Briefly, umbilical cord was disinfected by rinsing with 70% ethanol for 30 sec, and was then dissected with scissors into pieces around 1-3 mm^3^ in volume. These tissue pieces were plated in a cell culture dish under standard conditions using DMEM (Dulbecco’s Modified Eagle Medium) containing 10% fetal bovine serum (FBS) supplemented with 100 U/ml penicillin, and 100 µg/ml streptomycin (GIBCO, USA). Cell cultures were maintained in a humidified atmosphere with 5 % CO_2_ at 37^ o^C.

***Immunophenotyping***

To phenotype cell-surface antigens, fourth-passage cells were stained by conjugated antibodies specific for the following human antigens: CD90-FITC, CD44- FITC, and CD34- FITC, (BioLegend, USA) and compared to corresponding isotype control. Stained cells were analyzed using FACSCalibur flowcytometer (Becton Dickinson, USA). For each sample, at least 10,000 events were recorded (23).

Mesodermal differentiation: To investigate their capacity for mesodermal differentiation, adipogenic, and osteogenic differentiations were carried out by culturing cells with the differentiation kit (Bonyakhteh, Iran), and differentiation was evaluated by Oil-Red-O and Alizarin Red staining.

***Biochemical analysis***

The rats were anesthetized with an IP injection of ketamine (100 mg/kg) and xylazine (10 mg/kg). Then, blood samples were collected from all rats by heart puncture at the time of death. Blood samples were centrifuged at 3500 rpm at 25 ^°^C for 10 min, and serum was stored at −80 ^°^C for further assays. Serum levels of C-peptide were measured with enzyme-linked immunosorbent assay (ELISA) kit (RayBiotec, Norcross, Georgia). All samples were measured in duplicate. In addition, fasting blood glucose (FBG) in treatment groups were monitored daily by glucometer (ACCU-CHEK Performa, USA).

***Tissue harvesting***

*Brain*

In order to harvest brain tissue, the rats were decapitated and brains were removed immediately. The diencephalon was dissected out by an anterior coronal section, anterior to the optic chiasm, and a posterior coronal cut at the posterior border of the mammillary bodies. To separate ARC from anteroventral periventricular nucleus (AVPV), a third coronal cut was made through the middle of the optic tract, just rostral to the infundibulum (46). The specimens consisting of ARC and dorsomedial hypothalamic nucleus (DMH) were stored in liquid nitrogen for molecular study.

*Pancreas*

Pancreas tissues were harvested in control and diabetic rats for histology and in some treated groups on the 45^th^ day for immunohistochemistry (IHC) staining. The tissues were fixed overnight in a solution of freshly prepared 4% paraformaldehyde in 0.1 M PBS, pH 7.4, at 4 ^°^C. Samples were dehydrated and prepared as paraffin blocks, serially cut into 5 µm sections at 50 µm intervals, stained with hematoxylin and eosin (H&E) and examined by light microscopy. 

*Immunohistochemistry*

To detect insulin in pancreatic sections, primary anti-insulin antibody (1:100; DAKO, USA) were used. The appropriate primary antibody was added in blocking buffer and incubated overnight at 4 ^°^C. Sections were washed and incubated with secondary antibody for 30 min at room temperature, followed by washing and incubation with diaminobenzidine (DAB) as chromogen (Master Polymer Plus Detection System (Peroxidase), Granada, Spain) for 5 min. Sections were counterstained with hematoxylin for 2 to 5 min and mounted [43].

*RNA isolation and real time PCR*

Total RNA from 50-100 mg of brain tissue was isolated using TRIzol Reagent (Life Technologies, Rockville, Md, USA) according to the manufacturer’s instructions. The quantity of the extracted RNA was evaluated by Nanodrop (measuring the optical density 260/280), and the quality of RNA was assessed by running 3 μl on 1% agarose gel. The good quality was indicated by the lack of a smear and presence of 28S and 18S ribosomal RNA (rRNA). The total RNA was reverse-transcribed into cDNA using Prime Script RT Reagent Kit (Takara, Japan) according to the manufacturer’s guidelines. 

Primers were designed using Allele ID 7 and Oligo 7 software (Premier Biosoft International, Palo Alto, USA) for LEPR (NM_ 012596.1), melanocortin 4 receptor (MC4R*) *(NM_ 013099.3), NPY (NM_ 012614.2), and glyceraldehyde-3-phosphate dehydrogenase (GAPDH) (NM_ 017008.4). The rat *GAPDH* gene was used as the reference gene for data normalization (Table 1). The expression levels of MC4R, NPY, and LEPR were determined by Livak (2^-ΔΔCT^) method. Melt curves were also analyzed to confirm the specificity of reaction at the end of the program.

***Statistical analysis***

All statistical analyses were performed using SPSS version 19.0 software (SPSS Inc., Chicago, USA). The results were expressed as mean±SD values, and statistical significance was determined by one-way ANOVA with Tukey’s multiple comparison *post hoc* tests with *P*-value<0.05.

**Table 1:** Sequences of real time polymerase chain reaction (PCR) primers for evaluation of the relative expression of *MC4R*, *LEPR*, and *NPY* genes in rat

Primer	Sequence	Amplicon length (bp)
MC4R-F	5′-ACGGGTCAGAAACCATCGTC-3′	115
MC4R-R	5′-GCGAGCAAGGAGCTACAGAT-3^′^	
LEPR-F	5′-GCCATCAATTCCATCGGTGC-3′	131
LEPR-R	5′-GTCCAGGAAAGGATGACGCA-3′	
NPY-F	5′-CTGCGACACTACATCAA-3′	150
NPY-R	5′- CATTTCCCATCACCACAT-3′	
GAPDH-F	5′- AAAGAGATGCTGAACGGGCA-3′	100
GAPDH-R	5′-ACAAGGGAAACTTGTCCACGA-3′	

MC4R: Melanocortin-4 receptor; NPY: Neuropeptide Y; LEPR: Leptin receptor; GAPDH; Glyceraldehyde-3-phosphate dehydrogenase

**Figure 2:**
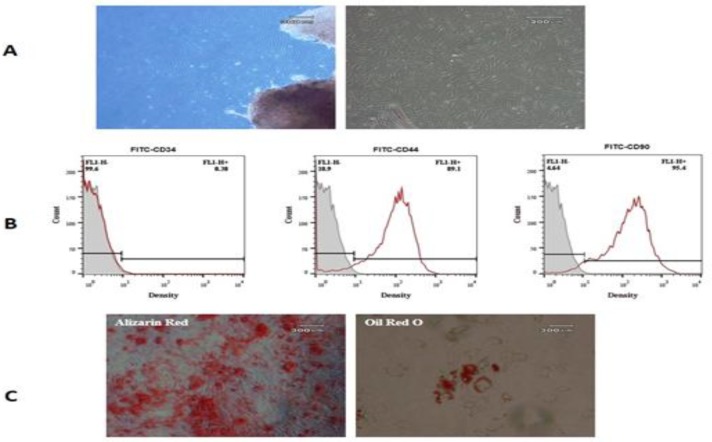
Characterization of human wharton's jelly-derived mesenchymal stem cells. (A) The morphology of mesenchymal stem cells (MSCs). (B) The result of flowcytometry showed that cells were negative for CD34 (0.3%) and positive for CD44 (89.1%) and CD90 (95.4%). (C) Differentiation of MSC into adipocytes and osteocytes that confirmed by oil red O and alizarin red, respectively

**Figure 3:**
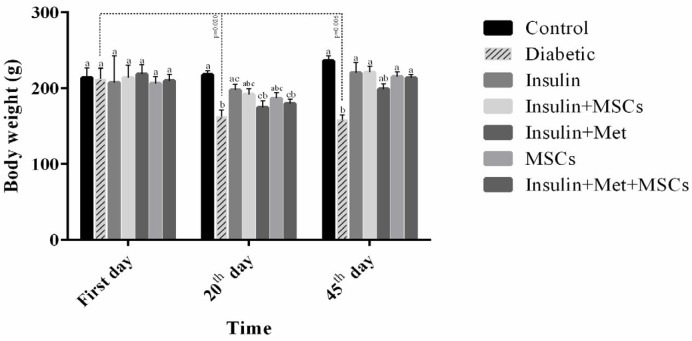
Body weight. There was no significant difference between columns, which have at least one similar letter. However, this dissimilar letters indicated a significant difference (*P*<0.05)

**Figure 4:**
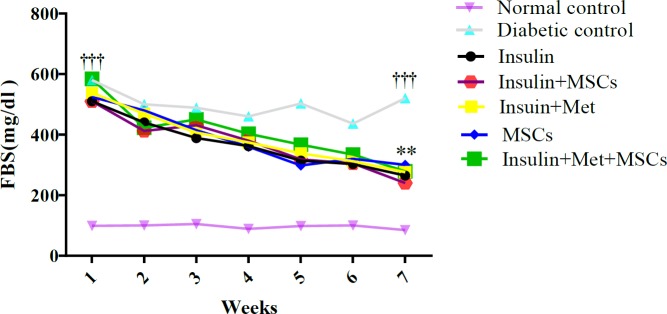
Changes in fasting blood glucose during the study. The result showed that changes in the blood glucose levels were slightly more decreased among the treatment groups than the diabetic group, but the changes were not significant between the treatment groups. The dissimilar asterisk indicated a significant difference between the diabetic group with the treatment groups (*P*-value<0.05)

**Figure 5:**
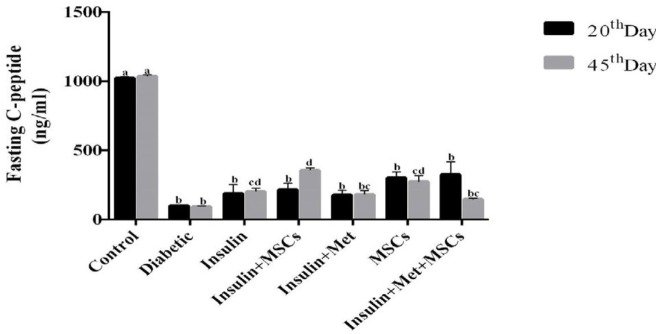
Changes in fasting C-peptide level between groups during the study. Levels of C-peptide on both of time were significantly more increased on the treatment groups rather than diabetic group. Changes were not significant in 20th days among the treatment groups, but on the 45^th^ day, C-peptide was significantly increased in the Insulin+MSCs group, Insulin. group, and MSCs group (*P*-value<0.05). a, b, c, and d: According to post hoc Tukey test that was used to make intergroup comparisons, groups with same superscripts were not significantly different at α=0.05 (*P*-value≥0.05). However, dissimilar letters indicate a significant difference (*P*-value <0.05). MSCs; Mesenchymal stem cells

**Figure 6:**
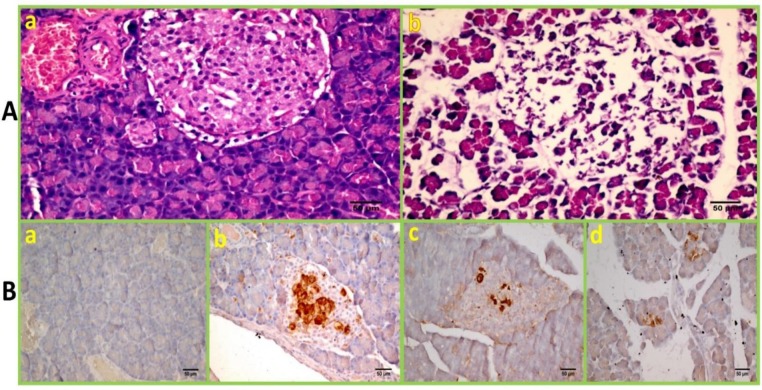
STZ-induced immunosuppressed diabetic mice. (A) Pancreatic sections of both a: normal and b: STZ-treated mice (H&E, ×40). (B) Pancreatic sections in different groups were immunostained for insulin (×40), a: diabetic, b: Insulin+MSCs, c: MSCs, d: Insulin+Metformin+MSCs groups. MSCs; Mesenchymal stem cells, STZ; Streptozotocin

**Figure 7:**
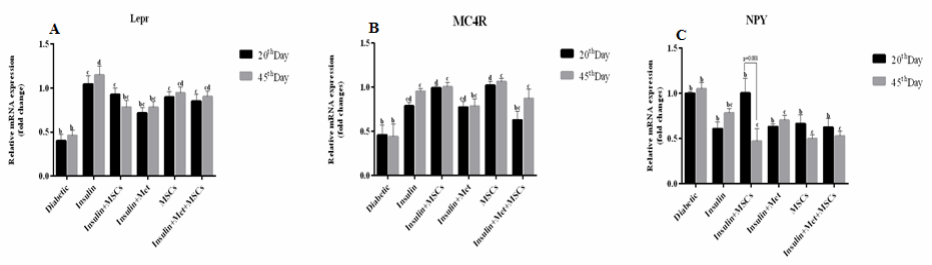
Expression of genes that control appetite and energy expenditure. (A, B) The expression level of LEPR and MC4R genes significantly up-regulated in the treatment groups (*P*-value<0.05). (C) The expression level of NPY gene significantly down-regulated in treatment group compared to diabetic group on the both time of study (*P*-value<0.05). a, b, c, and d: According to post hoc Tukey test that was used to make intergroup comparisons, groups with same superscripts were not significantly different at α=0.05 (*P*-value≥0.05). However, dissimilar letters indicate a significant difference (*P*-value<0.05). (LEPR; Leptin receptor, MC4R; Melanocortin-4 receptor, NPY; Neuropeptide Y)

## Results

***Characterization of WJ-MSCs***

The plastic-adherent cells from Wharton’s jelly formed a monolayer with fibroblast-like morphology (Figure 2A). The flow cytometry results demonstrated that the WJ-MSCs expressed stromal markers (CD90, CD44), while were negative for the hematopoietic marker (CD34) (Figure 2B.( After differentiation toward adipogenic and osteogenic lineage, the presence of lipid vacuoles was confirmed by Oil Red O; and calcium deposition revealed with Alizarin Red (Figure 2C).

***Animal general condition***

The high blood glucose levels (FBG ˃ 200 mg/dl) confirmed diabetic rats (three weeks after STZ injection). After 45 days of therapy, polydipsia, polyphagia, urorrhagia, and loss of body weight were ameliorated in the treatment groups compared to the diabetic group. The weight of rats on the 20^th^ and 45^th^ days after therapy were significantly decreased in the diabetics groups compared to other groups, while the weight of rats on the 20^th^ days in the treatment groups were decreased, but after this time were gradually elevated until the end of the study (Figure 3).

***Fasting blood glucose levels***

During the last period of study, the blood glucose levels in the diabetic group increased. Also, the blood glucose levels were more slightly decreased in the treatment groups than the diabetic group, but the change was not significant. The level of FBG in Ins. group has more reduction than other groups. Ins.MSCs, Ins.Met, and MSCs therapy groups exhibited similar decreasing trends and were almost close to the Ins. group. Although in Ins.Met.MSCs group the trend was observed, but overall the experiments showed a higher blood glucose level during the study (Figure 4).

***C-peptide levels***

Fasting C-peptide in all groups was examined and analyzed. There was no observed significant difference on the 20^th ^days in the therapy groups in comparison with the diabetic group, while on the 45^th^ day a significant increase in fasting C-peptide levels was observed in the Ins.MSCs, Ins., and MSCs groups (*P*-value<0.05) (Figure 5). 

***Histology and immunohistology***

A marked destruction in pancreatic islets was confirmed in STZ-treated animals (Figure 6A). The presence of insulin-producing β cells in treatment groups were evaluated by IHC. The Ins.MSCs group revealed the better results; the islet size was larger with more insulin-positive cells. In comparison, the MSCs group showed fewer islet clusters with less insulin-positive cells, and the Ins.Met.MSCs group had only few scattered insulin-positive cells between exocrine tissue. No insulin immunostaining cell was identified in the Ins.Met. group (Figure 6B).

***Expression of MC4R, NPY, and LEPR genes in brain tissues***

The expression of *LEPR* gene in ARC on days 20 and 45 was significantly increased in the Ins., Ins.Met.MSCs, and MSC groups compared to the diabetic group, but there was no significant difference in the Ins.Met. group than the diabetic group (Figure 7A). On the other hand, on day 20, *LEPR *gene expression was significantly increased in the Ins. group compared to the Ins.Met. and Ins.MSCs groups (*P*-value<0.05). The results in Figure.7B showed that *MC4R* gene expression on the 20^th^ and 45^th^ days was significantly increased in the Ins., Ins.MSCs, and MSC groups compare to the diabetic group (*P*-value <0.05). As well, on the 45^th^ days a significant increase in the gene expression was observed in the Ins.Met.MSCs group in comparison with the diabetic group.

The expression of *NPY* gene on the 20^th^ days was significantly decreased among the treatment groups than to the diabetic group, but there were no statistically significant differences. However, on the 45^th^ days, the expression level of *NPY* gene was significantly decreased in the Ins.Met., MSCs, and Ins.Met.MSCs groups compared to the diabetic group (*P*-value<0.05). Furthermore, statistically significant differences were observed in the Ins.MSCs, Ins.Met., MSCs, and Ins.Met.MSCs groups on the 20^th^ and 45^th^ days (Figure 7C).

## Discussion

T1D is a disease characterized by loss of insulin-producing β-cells as a result of an autoimmune-mediated destruction of Langerhans islet cells that leads to increased glucose and reduced C-peptide levels (45, 47). A usual therapy of T1D is whole organ or islet cells transplantation that has many limitations including lack of donors, rejections after transplantation, and requirement of long-term immune suppression (48). Recently, stem cells therapy has held immense promise for the treatment of patients with diabetes mellitus, from preclinical to clinical studies. Human MSC transplantations help to reduce blood glucose levels in diabetic animals and can improve blood glucose homeostasis in both type 1 and type 2 diabetic animals (49-51). In one study, a small proportion (6%) of diabetic MSCs expressed proinsulin and C-peptide, and these cells could differentiate rapidly into functional islet-like cells in the presence of a pseudo diabetic milieu (52). It has been suggested that the diabetic microenvironment may be a condition for MSC differentiation into insulin-producing cells *in vitro* and *in vivo. *

In other studies, labeled MSCs were transplanted into diabetic mice to determine their survival in body tissues. It was confirmed that the grafted cells presented and secreted functional human insulin and C-peptide (53, 54). A recent study by Maldonado *et al.* indicated that hUCWJCs after IP administration could migrate to damaged tissues and promote insulin secretion from non-pancreatic local cells (31). Similar previous studies in diabetic animal models have also shown that MSCs could alleviate blood glucose levels (53, 55, 56). Due to the absence of MHC class II and other co-stimulatory molecules on the surface of MSCs, they do not exhibit any immune response in the host tissue (15, 57).

In the present study, more insulin producing cells are present in the Ins.MSCs group, and it seems that treatment by Ins.MSCs may be better than cell therapy alone. Also, in our study, after the 20^th^ day of therapy, FBG level in treatment groups, especially in the Ins. and Ins.MSCs groups, was lower than the control group. Also, after 45 days, blood glucose levels in all treatment groups decreased compared to the control group. C-peptide is co-secreted with insulin on an equimolar basis from the pancreatic beta cell (58), it is believed that C-peptide concentration reflected changes in insulin secretion more accurately than insulin concentration alone (59). In our study, the level of C-peptide in the treatment groups increased compared to the diabetic group. This increase was also higher in the Ins.MSCs group than other groups, and there was significant difference between the Ins., Ins.MSCs and MSCs therapy groups compared to other treatment groups, which suggests the positive combination effect of MSCs and insulin on the improvement of diabetes status that is consistent with previous findings (53, 54). 

Metformin, an oral biguanide class of antihyperglycemic agent, is by far the most widely used glucose-lowering drug for type 2 diabetes mellitus (T2DM) (60). Besides common application in T2DM, metformin has been proved beneficial in patients with T1DM, due to the improvement of insulin sensitivity (61). 

In a study by Zhou *et al.*, metformin was shown to have a degenerative effect on beta cells (62), which reduces insulin secretion and subsequently reduces the amount of C-peptide. In our study, the C-peptide level was lower in the groups that received metformin. It seems that the adverse effect of metformin has been confirmed in our study (Figure 5). This finding is compatible with IHC result in the present study.

Glucose is the prime cellular energy source to keep up life, and therefore, its availability must be meticulously monitored (63, 64). The central nervous system (CNS) plays a crucial role in the regulation of energy and glucose homeostasis. The hypothalamus is one of the important regions involved in the central control of feeding and energy expenditure. The hypothalamus is constituted by distinct nuclei morphologically and functionally, including ARC, paraventricular (PVN), lateral hypothalamic area (LHA), ventromedial nucleus (VMN), and the dorsomedial nucleus (DMN), which all contain specific glucose-sensing neuronal populations (65, 66). In particular, the ARC of the hypothalamus is pivotal for the regulation of feeding and metabolism. Moreover, the ARC integrates hormonal and nutritional metabolic signal through the CNS. The ARC areas consisted of two antagonistic neuronal populations: one of them is the orexigenic (appetite-stimulating) NPY and AgRP, and the other is the anorexigenic (appetite-suppressing) including POMC and cocaine- and amphetamine-regulated transcript (67, 68). The overall balance between orexigenic and anorexigenic forces defines the final metabolic outcome (64). Moreover, ICV administration of insulin elevates the expression levels of POMC, which has an anorexigenic action and reduces the expression levels of NPY/AgRP that has an orexigenic action (69).

Previous studies have explained that insulin activates both LEP biosynthesis and secretion from white adipose tissue; LEP is able to improve metabolism, glucose and lipid levels in humans with T1D (70, 71). LEP is a peptide hormone with potent anti-diabetic actions that is produced and secreted by adipose tissue and relates to nutritional status (72). This hormone has important effects in the regulation of glucose homeostasis, food intake, and energy balance. LEP can alleviate diabetes via inhibition of the hypothalamic-pituitary-adrenal axis (73). These functions are mediated by actions on LEPR that is expressed by multiple neuronal groups in the CNS. Binding of LEP to LEPR triggers the activation of several intercellular pathways, including the Janus kinase/signal transducer and activator of transcription (68), mitogen-activated protein kinase, phoinositide 3-kinase (PI3K) (74), and mammalian target of rapamycin pathways (67, 75). LEP released from adipose tissue, go into ARC and acts on its receptors on two LEP-sensitive neuronal populations in ARC. One of them is POMC neurons, which expresses POMC that leads to the release of α-melanocyte stimulating hormone, a key intermediate of this pathway that acts on melanocortin receptors (MC3R/MC4R) and subsequently leads to reduced food intake, increased energy expenditure, ameliorating insulin sensitivity, and developing weight loss (76, 77). On the other hand, acute central delivery of MC4R agonists in mice causes a potent reduction in plasma insulin levels, which is not secondary to a lowering of blood glucose or food intake (78). Consistently, mice with genetic loss or overexpression of melanocortin signaling exhibit changes in circulating insulin, irrespective of body weight and feeding (78, 79). Another one is NPY/AgRP neurons in which the NPY and AgRP are as a melanocortin receptor blocker. Both cause to weight gain (65) and decrease energy expenditure (80, 81). In contrast to POMC neuron, the effect of LEP on NPY/AgRP neurons is inhibitory (36). Several circulating factors, including LEP and insulin, had been suggested to suppress the acute activity of NPY/AgRP neurons. The inhibitory effects of LEP and insulin in NPY/AgRP neurons have been directly associated with that of PI3K (82-87). Evidence also suggests a stimulatory effect of central NPY on insulin release. Ventricular delivery of NPY promotes insulin secretion and potentiates the insulinemic response to hyperglycemia, independently of food intake (88). Hypothalamic NPY secretion can be regulated by insulin hormone, and also the high level of insulin can inhibit the synthesis and release of NPY (71). 

In the present study, the expression of *LEPR* gene in the treatment groups showed that this increase in Ins. and MSC therapy groups was higher than other groups. Also, by increasing the level of LEP and subsequently the LEPR expression increment, the expression rates of MC4R increased and NPY decreased in the treatment groups, which was consistent with previous studies (89, 90). These changes were more prominent in the groups that received MSCs indicating the positive effect of cell therapy on reducing the expression of NPY and increasing the expression of MC4R, which follows an increase in appetite stimulation and gaining weight. 

Finally, the weight of mice in treatment groups in general increased at the end of study, but in groups treated with metformin this increase was lower than other groups that showed the side effect of metformin on weight loss. All in all, using WJ-MSCs in combination with insulin may modulate energy balance, and also these cells are favorite for short investigations because of low tumorigenesity and immunogenicity, but the use of them in the long term must be investigated in future studies. 

## Conclusion

Our study showed that infusion of WJ-MSCs and combined therapy of WJ-MSCs with insulin can significantly improve energy expenditure and weight gain in rats by changing the amount of LEPR and subsequent changes in the expression of NPY and MC4R. In future studies, it is suggested to follow the effects of these cells with different protocol, i.e. with more frequent intervals and lower doses, as well as synergistic effect of them with insulin on diabetic animal and preclinical study.
